# Immunomodulatory Effects of Adipose-Derived Stem Cells: Fact or Fiction?

**DOI:** 10.1155/2013/383685

**Published:** 2013-09-10

**Authors:** Angelo A. Leto Barone, Saami Khalifian, W. P. Andrew Lee, Gerald Brandacher

**Affiliations:** ^1^Vascularized Composite Allotransplantation (VCA) Laboratory, Department of Plastic and Reconstructive Surgery, Johns Hopkins University School of Medicine, Baltimore, MD 21205, USA; ^2^Reconstructive Transplantation Program, Department of Plastic and Reconstructive Surgery, Johns Hopkins University School of Medicine, Ross Research Building 749D, 720 Rutland Avenue, Baltimore, MD 21205, USA

## Abstract

Adipose-derived stromal cells (ASCs) are often referred to as adipose-derived stem cells due to their potential to undergo multilineage differentiation. Their promising role in tissue engineering and ability to modulate the immune system are the focus of extensive research. A number of clinical trials using ASCs are currently underway to better understand the role of such cell niche in enhancing or suppressing the immune response. If governable, such immunoregulatory role would find application in several conditions in which an immune response is present (i.e., autoimmune conditions) or feared (i.e., solid organ or reconstructive transplantation). Although allogeneic ASCs have been shown to prevent acute GvHD in both preclinical and clinical studies, their potential warrants further investigation. Well-designed and standardized clinical trials are necessary to prove the role of ASCs in the treatment of immune disorders or prevention of tissue rejection. In this paper we analyze the current literature on the role of ASCs in immunomodulation *in vitro* and *in vivo* and discuss their potential in regulating the immune system in the context of transplantation.

## 1. Introduction

Adipose-derived stromal cells (ASCs), often referred to as adipose-derived stem cells, are a type of mesenchymal stromal cells extracted from adipose tissue and have been described as fibroblast-like, adherent, and multipotent cells capable of multilineage differentiation. A number of clinical trials using ASCs are currently underway, including one to treat fistulas in patients affected by Crohn's disease [1, 2] as well as study on the treatment of calvarial defects [3]. Similarly, bone marrow-derived mesenchymal stromal cells (BM-MSCs) have gained wide recognition and international interest due to their potent immunomodulatory activity that has been assessed in different models, such as in reduction of graft-versus-host disease (GvHD) [4]. The immunomodulation that MSCs exert is due to a combined humoral and cellular effect, mediated both by cytokine expression/secretion and regulation of peripheral blood mononuclear cells (PBMCs) [5–9].

Similarly, ASCs have shown a cell dose-dependent inhibition of PBMCs proliferation that can be independent from cell contact [10, 11]. Recent studies have demonstrated low antigenicity of these cells and potent immunomodulatory effects of mesenchymal cell therapy in solid organ transplants, although studies in large animal models and more clinical trials are needed before the widespread use of such cells is possible in clinical transplantation [5]. Furthermore, several recent studies are investigating their use for the treatment of autoimmune or degenerative diseases, as well as in reconstructive transplantation [12].

The relevance and potential of ASCs in tissue engineering and, potentially, in the treatment of “immune-mediated” conditions may be better appreciated when the stem cell yield of adipose tissue is examined. A single liposuction procedure may produce liters of fat, which are typically discarded. However, just one milliliter of this fat is enough to produce 250,000 ASCs in a single passage, and more than 1 billion cells by passage 3 or 4 [13, 14]. In less than a month, these cells can expand an additional 64-fold [15]. Thus, if all of the fat from a liposuction procedure is used, one can generate billions or trillions of stem cells with relatively minimal culture time. This is a significant advantage of ASCs, as the quality of mesenchymal stem cells (MSCs) in general is compromised when exposed to long-term culture. Specifically, prolonged culture of MSCs has been shown to limit differentiation potential [16] and proliferative capacity [17]. Furthermore, BM-MSCs undergo senescence with prolonged tissue culture, a phenomenon that has not been observed in ASCs [18]. The ease of harvest, genetic stability, and proliferative capacity of ASCs synergize to provide these cells with a significant advantage over other types of stem cells. 

Here we will review the current literature on the role of ASCs in immunomodulation *in vitro* and *in vivo* and discuss the role that cell passaging and upstream progenitors may play in the regulation of the immune system. 

## 2. Immunosuppression or Immunostimulation?

Ample evidence exists demonstrating the immunomodulatory capabilities of every class of MSCs [19]. Although MSCs can be derived from adipose tissue, bone marrow, umbilical cord blood, and Wharton's jelly, for the purpose of this review we will focus primarily on ASCs. Functional characterization of BM-MSCs and ASCs has shown that both cell types do not express HLA-DR (of the major histocompatibility complex class II, MHC II), which renders these cells significantly less immunogenic than other cell types, while they are also capable of suppressing lymphocyte reactivity in mixed lymphocyte reaction (MLR) assays and reducing inflammation *in vivo* [8, 15, 20–23]. Specifically, ASCs have been shown to inhibit the production of inflammatory cytokines and stimulate the production of anti-inflammatory cytokines and antigen-specific Treg cells [24]. These immunosuppressive properties together with their immunoprivileged status [24, 25] allow ASCs to be used in allogeneic and xenogeneic transplants without the need for immunosuppressants [20, 26]. Additionally, ASCs augment tissue repair, promote angiogenesis, and limit apoptosis, which poise these stem cells to make significant contributions to regenerative medicine, especially since large quantities can be harvested through minimally invasive techniques [20]. 

The immunomodulation exerted by ASCs can produce immunosuppressive or immunostimulatory effects. The immunosuppressive properties of ASCs have been consistently demonstrated in a variety of studies both *in vitro* and *in vivo* [14, 19, 27, 28]. On the other hand, the immunostimulatory response of ASCs is a dynamic process that may change with subsequent passages while in culture. For example, early passage ASCs promote a proliferative response of allogeneic responder T cells in MLRs, whereas ASCs actually suppress the proliferative response beyond passage P1 [14]. This decline in immunostimulation after the initiation of culture can be explained, in part, by the presence of antigen presenting cells (APCs) within the population [29]. Studies have shown that early passages of ASCs express markers, such as MHC II, CD45, CD80, and CD86, which stimulate APCs and trigger the immune response [14]. However, with continued passaging of ASCs, these APC-associated markers are lost, effectively mitigating the immune response until it has been eliminated. Thus, the immunophenotypic changes of ASCs are directly correlated with their ability to act as stimulator cells. 

The precise immunomodulatory properties of ASCs that is the extent to which they augment or downregulate the immune response are a matter of great debate. Several considerations fuel the controversy regarding the immunostimulatory or immunosuppressive effects of ASCs. These include (1) the inability of *passaged* ASCs to stimulate an allogeneic immune response [14], (2) the role of soluble factors and cell-cell contact in promoting an immunosuppressive effect [11, 19, 30], and (3) the potential for tumor development and/or growth [29, 31, 32]. As outlined above, one hypothesis for the immunosuppressive effect of passaged ASCs is the loss of APC-associated markers. On the other hand, ASCs may inherently have low immunogenicity or may be susceptible to lysis by CD8^+^ T cells and NK cells [14, 33]. An alternative hypothesis is that the expression of soluble factors mitigates an active T cell response [11]. These soluble factors include, amongst others, prostaglandin E2 [23, 34], indoleamine 2, 3 dioxygenase (IDO) [28], hepatocyte growth factor [35], and leukemia inhibitory factor [36]. Several studies have shown that the specific inhibition of each of these soluble factors has nullified the immunosuppressive effects of ASCs. Additionally, expression of cytokines such as IL-6 appears to downregulate expression of MHC-II and CD86 on dendritic cells and inhibit their differentiation, further suppressing the immune response by hampering antigen presentation and/or costimulatory signaling of APCs. However, the data regarding the requirement of cell-cell contact is less clear. Some studies have demonstrated that such contact is necessary, whereas others have shown opposing evidence that ASCs can exert an immunosuppressive effect without cell-cell contact [11, 23, 30]. 

It is known that a fully competent immune system is necessary for early detection and destruction of tumor cells. For this reason, due to the immunomodulatory activity that ASCs may exert, when discussing the use of ASCs in humans the most troubling aspect of the debate is the potential for ASCs to stimulate tumor growth. Although there have been studies demonstrating that the immunosuppressive effects of ASCs are associated with the development of tumors, other groups have refuted these findings [31, 32, 37]. For example, local and systemic injections of ASCs were shown to induce homing of such cells to existent tumors and augment proliferation of tumor cells in mice [37]. Similarly, allogeneic stem cell injections into a patient with ataxia telangiectasia subsequently led to the development of a tumor, and tissue analysis confirmed tumor cells to be of donor origin [38]. On the other hand, transgene-expressing ASCs exerted an effective antitumor effect [31] and Cousin et al. demonstrated that this antitumor effect is mediated by ASCs interfering with cell cycle progression [39]. In fact, this group showed that ASCs strongly inhibited the proliferation of pancreatic ductal adenocarcinoma cells *in vivo*. Nevertheless, the role of ASCs in tumor biology remains to be fully elucidated.

## 3. Immunomodulation *In Vitro*

The immunomodulatory potential of ASCs abound as they have the capacity to differentiate into various types of mesenchymal tissue. *In vitro* experiments have verified that ASCs can differentiate into fat, muscle, cartilage, and bone, which engender significant implications for the therapeutic role of these cells in the future [40–44]. Cultured ASCs also contain progenitor cells that support angiogenesis by differentiating into endothelial cells [43, 45, 46]. Typically, ASCs can be found in the capillary walls and microvasculature of adipose tissue; thus, they may inherently play a role in the development of this vascular framework [47]. Indeed, the participation of ASCs in angiogenesis stabilizes endothelial networks and serves as a foundation for various vascular structures [48]. 

Cytokines and trophic factors are critical in mediating the immunomodulatory properties of ASCs. Interestingly, there appears to be no significant difference between the release of these factors between ASCs and other types of MSCs [19]. Key cytokines involved in modulating the immune response of ASCs include TNF-*α*, IFN-*γ*, IDO, and IL-17. Particularly, TNF-*α* has been shown to increase the immunosuppressive effect of ASCs by significantly enhancing their production of prostaglandin-E2 [19]. IFN-*γ* contributes to the immunomodulatory effects of ASCs by directly inducing the production of IDO [49, 50]. IDO inhibits T cell proliferation, which manifests as the immunosuppressive aspect of ASCs often noted in MLRs [51]. Thus, the interplay between ASCs and T cells is mediated by these cytokines, as activated T cells produce TNF-*α* and IFN-*γ*, which regulate the immunomodulatory functions of ASCs. In turn, ASCs suppress the proliferative response of T cells and can effectively reduce the amount of TNF-*α* and IFN-*γ* produced by activated T cells [52]. Notably, the composition of the cytokine milieu changes with subsequent passages of ASCs. For example, coculturing ASCs from passage 3 with PBMCs of a patient with SLE decreased the number of present Th17 cells, which suppressed the production of the proinflammatory cytokine IL-17. However, ASCs from passage 8 actually increased the proportion of Th17 cells and thus led to a proinflammatory state with increased expression of IL-17 [26]. From these experiments, it can be deduced that ASCs, like BM-MSCs, are not immune from the effects of prolonged culture. 

The shared characteristics of ASCs and BM-MSCs extend beyond their response to culture conditions. Indeed, ASCs can suppress the alloimmune response and proliferation of T cells as effectively as BM-MSCs [14, 19]. Both cell types lack MHC-II expression, primarily mediate their immunosuppressive effects via prostaglandin E2, and displayed a concentration-dependent immunosuppressive effect when added to MLRs [14, 26]. Further studies demonstrated that allogeneic ASCs and BM-MSCs actually stimulated a lesser proliferative response than allogeneic PBMCs and that these cells changed their immunophenotype in a similar way after multiple passages [8, 15, 53–55]. These findings suggest that ASCs may potentially replace BM-MSCs in the field of transplantation [19]. 

## 4. Immunomodulation *In Vivo*

The use of ASCs for tissue repair and replacement has precipitated numerous preclinical and clinical studies attempting to elucidate the therapeutic potential of these cells. Here we will report on recently completed preclinical studies followed by case reports and clinical trials. 

The profound immunomodulatory impact of ASCs has already been demonstrated in a variety of experimental models of disease, including spinal cord injury, neurodegenerative diseases, autoimmune diseases, and GvHD. 

The effect of ASCs on the central nervous system has also been demonstrated in a mouse model of Alzheimer's disease (Tg2576) [56]. Intravenous injection of hASCs significantly improved the memory, learning, and pathology of Tg mice for up to four months after injection and decreased the number of amyloid plaques compared to Tg-sham mice. This data supports the hypothesis on their ability to cross the blood brain barrier to reach the brain, and that ASCs can upregulate IL-10 and VEGF, which may have clinical implications for the treatment of this disease in the future. 

Systemic infusion of hASCs has also been shown to improve autoimmune hearing loss in a mouse model [57]. Injections of ASCs led to protection of hair cells and significantly improved hearing function in these mice. Again, the role of IL-10 was demonstrated via an upregulation of IL-10 secretion from Tregs. IL-10 suppresses the autoreactivity of T cells, which helps maintaining self-tolerance and inhibits the Th1 and Th17 responses, thereby decreasing production of the proinflammatory cytokine IL-17. 

Another major application of ASCs is in the field of transplantation. Allogeneic ASCs have been shown to prevent acute GvHD in both preclinical and clinical studies [26, 29, 58–61]. Transplantation of allogeneic ASCs controlled GvHD in a mouse model [62] and was subsequently shown to successfully treat GvHD in 7 out of 9 patients [59–61]. It is likely that the mechanism responsible for these improved outcomes is a shift from a proinflammatory to anti-inflammatory cytokine milieu, including IFN-*γ* [26]. Notably, allogeneic ASC transplantation also led to resolution of refractory red cell aplasia in patients after major ABO-incompatible HSC transplantation [58]. Because ASCs suppress alloreactive T cell proliferation, these cells can be transplanted across MHC barriers without stimulating an immune reaction. Accordingly, the infusion of ASCs with donor organs or VCA may enhance engraftment and reduce or eliminate GvHD [19]. In the past, BM-MSCs have been shown to prolong survival of allografts in experimental large and small animal models of reconstructive transplantation [63, 64]. More studies investigating the role of ASCs in prolongation of VCA survival or tolerance induction in large animal models are needed.

Results from clinical trials have confirmed the safety and efficacy of ASCs in treating a variety of diseases, including diabetes mellitus (DM), rheumatoid arthritis (RA), ulcerative colitis, fistulas, and other soft-tissue defects [2, 20, 24, 25, 65]. Diabetic patients infused with bone marrow and ASCs had a 40% decrease in insulin requirements and up to 26-fold increase in serum c-peptide levels with no adverse effects in a 3-month follow-up period [29]. Studies evaluating the therapeutic impact of ASCs in patients with diabetic mellitus foot showed improved rest pain score, walking time, and evidence of increased vascular collateral networks within 6 months of intramuscular ASC injection [20]. In RA, ASCs have been shown to regulate self-tolerance by suppressing the T cell and inflammatory response and play a role in activating Tregs, effectively reducing the severity of the disease in patients [24]. This dual immunomodulatory effect has been observed both *in vitro* and *in vivo* [26]. Similarly, infusion of ASCs reduced the severity of colitis in patients with longstanding disease, as evidence by increased survival and decreased diarrhea, inflammation and weight loss [25]. Finally, ASCs in conjunction with fibrin glue were found to be superior to fibrin glue alone in the treatment of complex perianal fistulas [1]. 

 The success of ASCs in clinical trials illuminates the potential impact that these cells may have on a wide spectrum of human disease. However, inherent variability exists in the quality and quantity of ASCs that are harvested due to donor age, disease status, body mass index (BMI), and harvest site [20, 21]. These idiosyncrasies will require further studies to elucidate what role, if any, they play in outcomes after ASC transplantation. 

## 5. An Alternative to Traditional ASCs: A Step Forward? 

The dictions “Adipose-derived Stem Cells” and “Adipose-derived Stromal Cells” have been interchangeably used since the description of this cell niche. However, while in the first definition lies the intrinsic characteristic of stem cells to undergo multipotent differentiation toward the mesenchymal lineages, the second confers more importance on the origin of such cells, from the stromal part of connective tissue derived from the mesenchyme. Additionally it depicts them as cells found in the supportive structure in which the functional cells of the adipose tissue reside, without posing any strong statement on their definition as “true” stem cells. In 2005, the International Society for Cellular Therapy (ISCT) has stated that multipotent mesenchymal stromal cells are the recommended designation for the cells often labeled as mesenchymal stem cells [66]. As more studies are performed and differences between MSCs from different tissues are emerging as these cell niches are investigated, specific challenges in determining an appropriate definition arise. Dominici et al. have proposed three criteria to define MSCs: (i) adherence to plastic; (ii) specific surface antigen (Ag) expression (CD105, CD73, and CD90) or absence (CD45, CD34, CD14, or CD11b, CD19, HLA class II); and (iii) multipotent differentiation potential (toward the osteogenic, chondrogenic, and adipogenic lineages) [67].

Our group agrees with applying this terminology to the adherent multipotent cells extracted from fat tissue, by using the more wide and nonspecific terminology of adipose-derived stromal cells. In the past, some groups have been able to maintain and expand cells extracted from adipose tissue also in nonadherent conditions but considered these cells as the same cell population (ASCs) that were isolated and cultured using different culturing conditions [68, 69]. However, as studies from the Palermo group have shown, upstream stem cell progenitors from different tissues display lack of adhesion and growth as floating spherical aggregates [70, 71]. When cultured in suspension, a cell population within adipose tissue maintains most of the stem cell biological characteristics and, if plated under differentiating conditions, undergoes adherence and change in their surface molecule expression (manuscript submitted). For this reason, studies undergoing in our laboratory are currently aiming to investigate the phenotypic and intrinsic biological differences between adherent cells and nonadherent cells. We have preliminary evidence that the ability to grow nonadherent cells derived from adipose tissue may represent the epiphenomenon of the observation of a more undifferentiated cell niche with biological and phenotypical unique characteristics, and that such cells may represent upstream progenitor cells in adipose tissue [72]. 

We are currently investigating the ability of this nonadherent cell fraction to modulate the immune response in a simulated* in vitro *transplant setting to assess the potential of such an upstream cell niche to act as enhancers or silencers of cellular responses. Although only preliminary data is currently available to reach a significant conclusion, the vision of a stronger immunomodulatory effect of this upstream cell niche warrants further investigation as it, if found, may represent a possible readily-available and abundant stem cell progenitor alternative to bone marrow for cell-based tolerogenic regimens. Conversely, the evidence of such cells stimulating the immune system may provide preliminary *in vitro *evidence of possible future applications of these cells in medical conditions that may require an immunostimulatory effect. Moreover, further studies assessing eventual shifts of their effect on the immune system depending on their concentration (as observed in MLRs plated with ASCs at different concentrations) [30] will be paramount.

Whether these upstream cells will, indeed, prove useful in any field besides tissue engineering (i.e. transplantation immunology or cancer monitoring) is yet to be fully investigated, and more studies to address such questions are necessary.

## 6. Immunomodulation in VCA: A New Horizon?

The use of efficacious cell-based approaches that may allow reduction or elimination of long-term immunosuppression has been the focus of scientific interest in the field of transplantation over the past decades. Along those lines, the application of ASCs in VCA is currently being investigated and may one day eliminate the need for complex and/or lifelong immunosuppression regimens that are currently an impediment to the widespread application of this therapeutic modality. The ability to obtain autologous ASCs and readily prepare them for injection into VCA patients for immunomodulatory purposes is a major contributing factor supporting the use of ASCs. As previously mentioned, as little as one gram of autologous fat can be expanded into millions of cells that can subsequently be used to improve VCA outcomes. Studies have shown that angiogenic factors appear to promote VCA outcomes as was demonstrated by revascularization of ischemic hind limbs in mice after injection of hASCs [35, 73, 74]. Importantly, the use of human ASCs was found to be superior to human BM-MSCs at salvaging ischemic hind limbs, further promoting the use of ASCs in this setting [74]. This may be due to superior survival, engraftment, and *in vivo* regenerative potential of ASCs compared to other types of MSCs. 

Similarly, local injection of ASCs has been shown to support wound healing in rat and swine models [75, 76]. Autologous ASCs improved epidermal healing and survival in full thickness skin grafts and was shown to rescue ischemic regions of skin flaps. Topical administration of ASCs has also been shown to be effective in murine models. Wound healing was accelerated in rats and diabetic mice with skin ulcers when ASCs were delivered in topical form [77, 78]. This has clinical implications for VCA, as wound healing may be compromised by the immunosuppressive regimens used after transplantation. 

A major concern for patients after transplantation of composite tissue is acute GvHD. Intravenous injection of ASCs in organ transplant recipients completely resolved acute GvHD in 83% of patients with median followup of 40 months [59]. Specifically in liver transplant recipients, infusion of unrelated ASCs also decreased the incidence of acute GvHD [60, 61]. Studies on the use of MSCs together with BMT and cyclosporine A in a swine hind limb VCA model demonstrated allograft survival for more than 200 days without signs of GvHD [63]. Recipients of this treatment protocol had no evidence of rejection in donor skin or muscle biopsies. The sum of these findings suggests that ASCs may be a promising immunomodulatory strategy in the setting of both VCA and solid organ transplantation to induce tolerance, prevent rejection, and rescue ischemic tissues. 

## 7. Immunomodulatory Properties of ASCs: Fact of Fiction?

As we collect more scientific evidence on the possible clinical applications of traditional ASCs, we are also witnessing at the same time an increasing number of reports of clinical treatments performed without the oversight of national regulatory bodies that are not published in peer-reviewed journals. The widespread concept of administering any kind of MSCs systemically to allow localization at the site of tissue injury clashes with the accepted newer concept of a paracrine and anti-inflammatory effect of such cells. The lack of rigorous monitoring of several parameters (i.e., administered dose, timing, and route of cell administration) together with the confounding bias introduced by simultaneous administration of anti-inflammatory drugs may also be responsible for the suboptimal study outcomes [79]. 

The caution that we need to use when evaluating these studies, however, should not preclude one from recognizing promising results obtained from several phase II clinical trials, for example, those that used MSCs to treat steroid-resistant acute GvHD [4]. Such encouraging observations warrant further investigation, ideally through prospective randomized controlled trials. Preclinical data have also shown encouraging results on the regenerative effect of MSCs in the reduction of ventricular arrhythmias and improvement of pulmonary function in patients affected from acute myocardial infarction [80]. Appropriate and safe labeling of ASCs and MSCs for cell tracking within tissues will be necessary to clearly determine the correlation between stem cells presence within the tissue and clinical outcomes. In addition, we agree with Keating on the need to establish a database registry of cell therapy recipients in order to keep record of clinical outcomes while monitoring for the presence of long-term adverse events [79]. Lastly, in-depth investigation of the clinical implications that chromosomal aberrations observed in cultures of MSCs *in vitro* may have once applied in the clinic [81, 82], and the potential interactions of MSCs with cancers is warranted, due to the controversial current scientific evidence on the topic. 

## 8. Conclusions

Despite increasing evidence that shifts the balance between “fact or fiction” to indicate that such potent immunomodulatory properties *in vitro* exist, translation of these findings to the *in vivo *scenario for ASCs is still challenging. As we await for well-designed clinical trials to prove the role of ASCs in the treatment of tissue injury or immune disorders, an important role will be played by basic-science research investigating any difference in cell phenotype, genotype, or biologic behavior depending on different parameters. Such parameters, amongst others, should include: (a) the culture conditions (adherent versus nonadherent), (b) oxygen tension, (c) differences between freshly-isolated and freeze-thawed cells, and (d) autologous versus allogeneic cells. Only when all these parameters will be validly assessed and outlined, standardization of MSCs therapy will be possible and the introduction of such cell-based approached introduced stably in controlled clinical practice. As Cicerone stated “In every art, or study, or any science, as in virtue itself, perfection is extremely rare.” Despite the wise and still current concepts highlighted from this aphorism, as physician scientists we need to acknowledge the “imperfect” (variable) biological responses and make it our duty to investigate the field further to obtain safe, effective, and reproducible therapeutic effects.
